# From Gateway to MultiSite Gateway in one recombination event

**DOI:** 10.1186/1471-2199-7-46

**Published:** 2006-12-06

**Authors:** Enrico Magnani, Linnea Bartling, Sarah Hake

**Affiliations:** 1 Department of Plant and Microbial Biology, University of California, Berkeley, California 94720, USA; 2 Plant Gene Expression Center, United States Department of Agriculture-Agriculture Research Service, Albany, California 94710, USA

## Abstract

**Background:**

Invitrogen Gateway technology exploits the integrase/*att *site-specific recombination system for directional cloning of PCR products and the subsequent subcloning into destination vectors. One or three DNA segments can be cloned using Gateway or MultiSite Gateway respectively. A vast number of single-site Gateway destination vectors have been created while MultiSite Gateway is limited to few destination vectors and therefore to few applications. The aim of this work was to make the MultiSite Gateway technology available for multiple biological purposes.

**Results:**

We created a construct, pDONR-R4-R3, to easily convert any available Gateway destination vector to a MultiSite Gateway vector in a single recombination reaction. In addition, we designed pDONR-R4-R3 so that DNA fragments already cloned upstream or downstream of the Gateway cassette in the original destination vectors can still be utilized for promoter-gene or translational fusions after the conversion.

**Conclusion:**

Our tool makes MultiSite Gateway a more widely accessible technology and expands its applications by exploiting all the features of the Gateway vectors already available.

## Background

Recombinase-based cloning technologies are becoming increasingly popular because of their easy use and high efficiency. These tools exploit bacterial or viral site-specific recombinases like the bacteriophage P1 Cre, the *Saccharomyces cerevisiae *FLP or the bacteriophage lambda integrase [1-3]. These enzymes catalyze a reciprocal double-stranded DNA exchange between two specific DNA sites [4].

**Figure 1:**
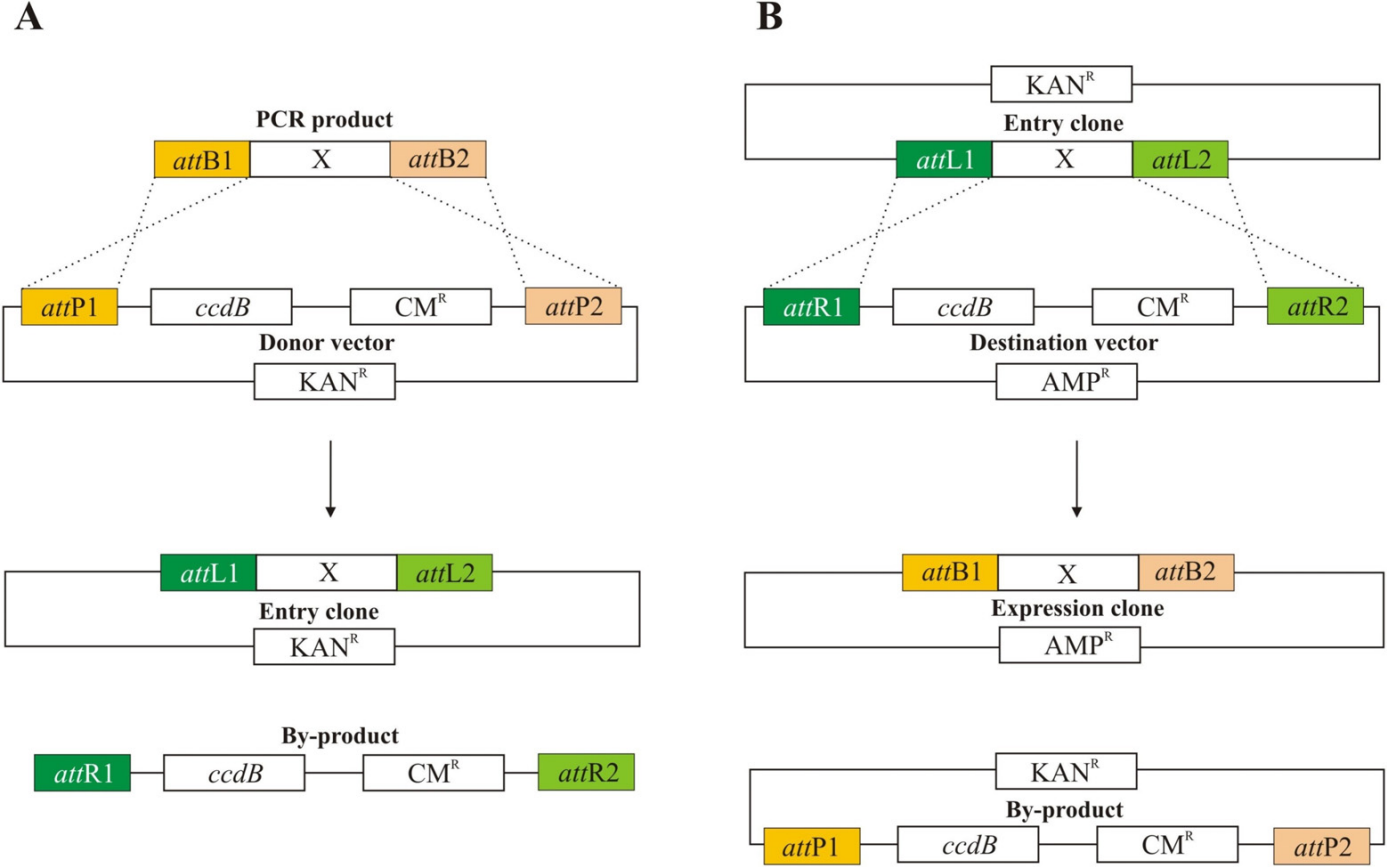
**Gateway BP and LR reactions**. (**A**) BP recombination of a PCR product "X" flanked by *att*B sites with a Gateway donor vector [3]. (**B**) LR recombination of an entry clone bearing a DNA fragment "X" with a Gateway destination vector [3].

Gateway (Invitrogen) is one of the most popular recombination cloning technologies [3]. It is based on the *Escherichia coli *bacteriophage lambda integrase/*att *system [5]. Two sets of reactions are employed in this technology: LR and BP recombinations (Fig 1). The BP reaction is catalyzed by the BP Clonase enzyme mix (Invitrogen), which recombines *att*B sites with *att*P sites. The LR Clonase mix (Invitrogen) is responsible for recombination of *att*L sites with *att*R sites. Any DNA fragment of interest can be PCR amplified with primers containing *att*B sites and cloned into a donor vector carrying *att*P sites in the presence of the BP Clonase mix. The recombination of *att*B with *att*P sites leads to the formation of *att*L and *att*R sites. The reaction creates an entry clone bearing the insert of interest flanked by *att*L sites and a byproduct flanked by *att*R sites (Fig 1A). The insert can then be mobilized into any destination vector having *att*R sites via an LR reaction (Fig 1B). The resulting construct can be easily selected using a combination of a positive (antibiotic resistance) and a negative (the cytotoxic *ccdB *gene) selection which does not allow the growth of *E. coli *transformed with either the initial vectors or the by-product [3,6]. The vast number of Gateway destination vectors designed for many different biological analyses and the large collections of entry clones carrying entire genome open reading frames make this technology highly attractive.

**Figure 2:**
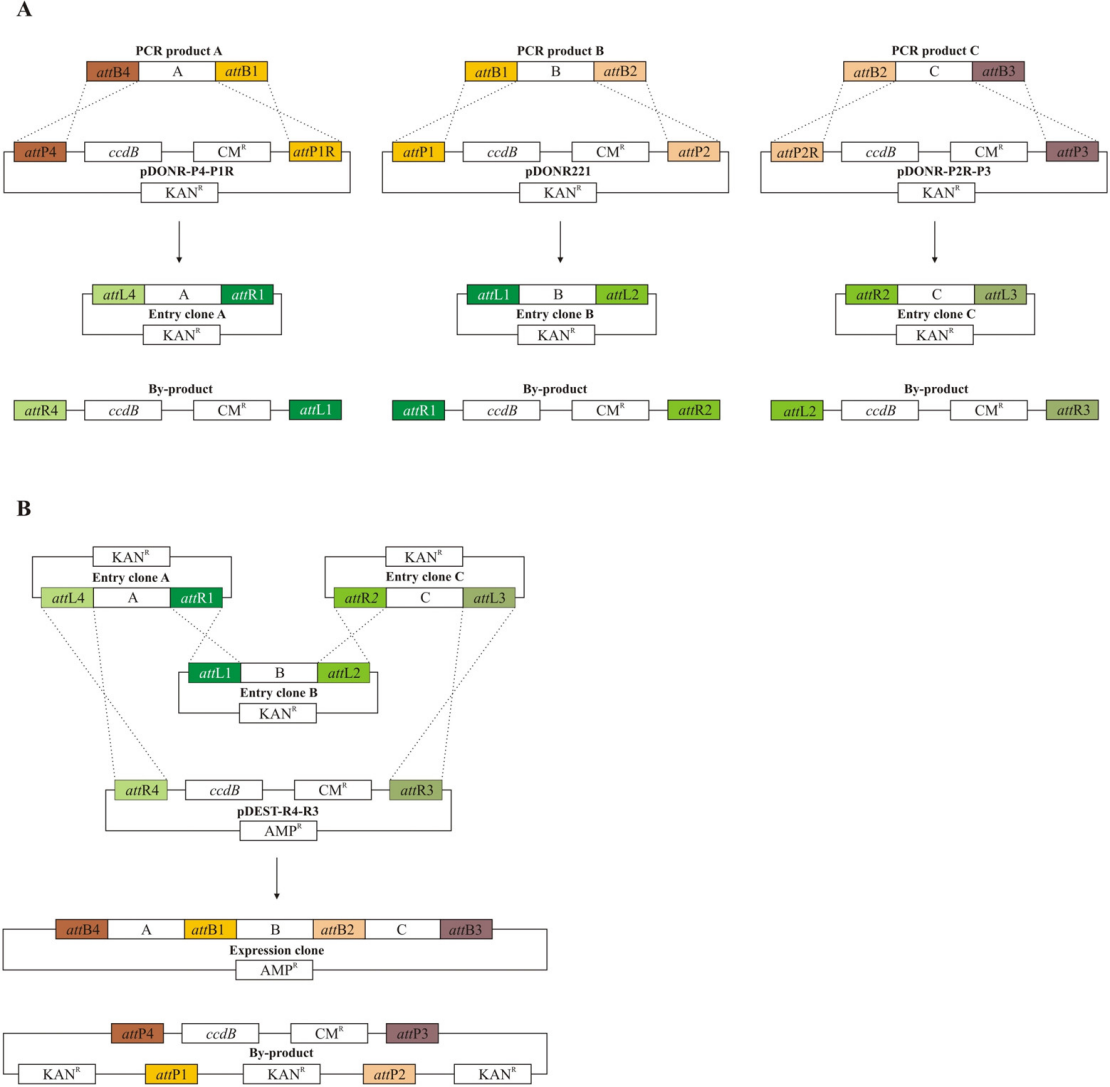
**MultiSite Gateway**. (**A**) BP recombinations of three PCR products "A", "B" and "C" flanked by *att*B sites with three Gateway donor vectors [7]. (**B**) LR plus recombination of three entry clones bearing the DNA fragments "A", "B" and "C" with the MultiSite Gateway destination vector pDEST-R4-R3 (Invitrogen) [7].

A variant of Gateway, named MultiSite Gateway (Invitrogen), has been developed to facilitate the cloning of multiple DNA fragments [7]. By using four different *att*B sites (*att*B1, *att*B2, *att*B3, and *att*B4), three PCR products flanked by specific *att*B sites can be cloned into three donor vectors bearing the corresponding *att*P sites (Fig 2A). The LR Clonase Plus mix (Invitrogen) specifically recombines all *att*L sites with their corresponding *att*R sites. A single LR Plus reaction with the three entry clones and the pDEST-R4-R3 destination vector (Invitrogen) creates a construct with the three inserts in a defined and oriented order (Fig 2B). Any Gateway entry clone can be mobilized into a MultiSite Gateway destination vector. Compared to Gateway, MultiSite Gateway is limited by the availability of a small number of destination vectors which prevents this powerful technology from being used for multiple biological purposes.

Here we present a method to convert any Gateway vector to a MultiSite Gateway vector via a single recombination event.

## Results and discussion

**Figure 3:**
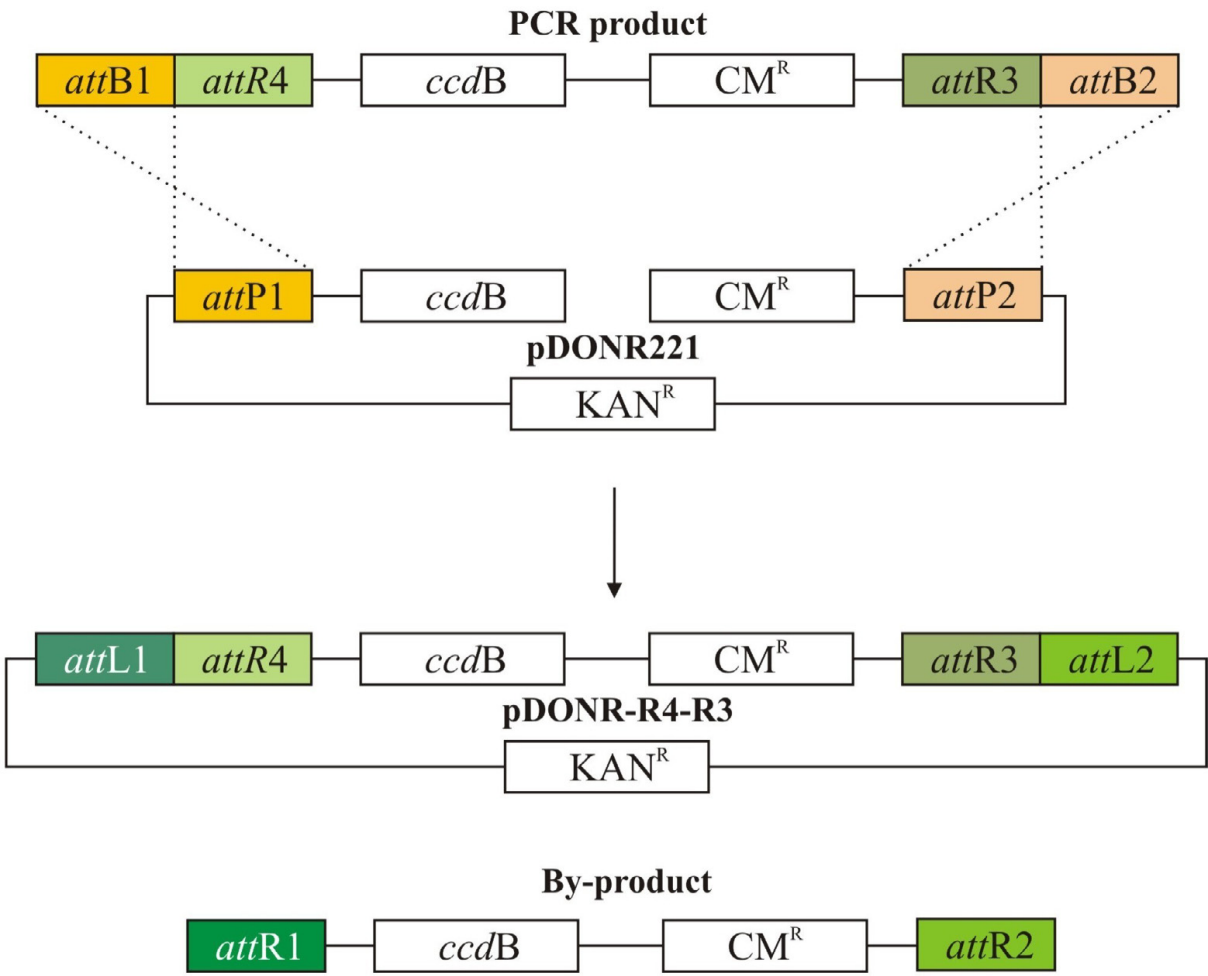
**pDONR-R4-R3**. BP recombination of the *att*R4-*att*R3 Gateway cassette flanked by *att*B1 and *att*B2 sites with a linear Gateway donor vector pDONR221 (Invitrogen) which leads to the creation of the vector pDONR-R4-R3 and a cytotoxic by-product.

We decided to take advantage of the large number of Gateway single-site destination vectors and developed a new method to easily convert them to MultiSite Gateway. The functional part of MultiSite Gateway destination vectors is the *att*R4-*att*R3 cassette which can recombine with three Gateway donor vectors carrying the appropriate *att*L sites (Fig 2B) [7]. We constructed a vector, pDONR-R4-R3, to mobilize the *att*R4-*att*R3 cassette into any Gateway Destination vector. To create pDONR-R4-R3, we cloned a Gateway cassette inside another Gateway cassette by choosing a combination of *att *sites that cannot recombine with one another. We exploited the ability of the BP Clonase enzyme mix to recombine *att*B sites with *att*P sites while leaving *att*R sites untouched [3]. The *att*R4-*att*R3 cassette was amplified using *att*B primers and cloned into pDONR221 (Invitrogen), a vector carrying an *att*P1-*att*P2 cassette. The BP recombination created the pDONR-R4-R3 vector that contains the *att*R4-*att*R3 cassette flanked by *att*L1-*att*L2 sites (Fig 3).

**Figure 4:**
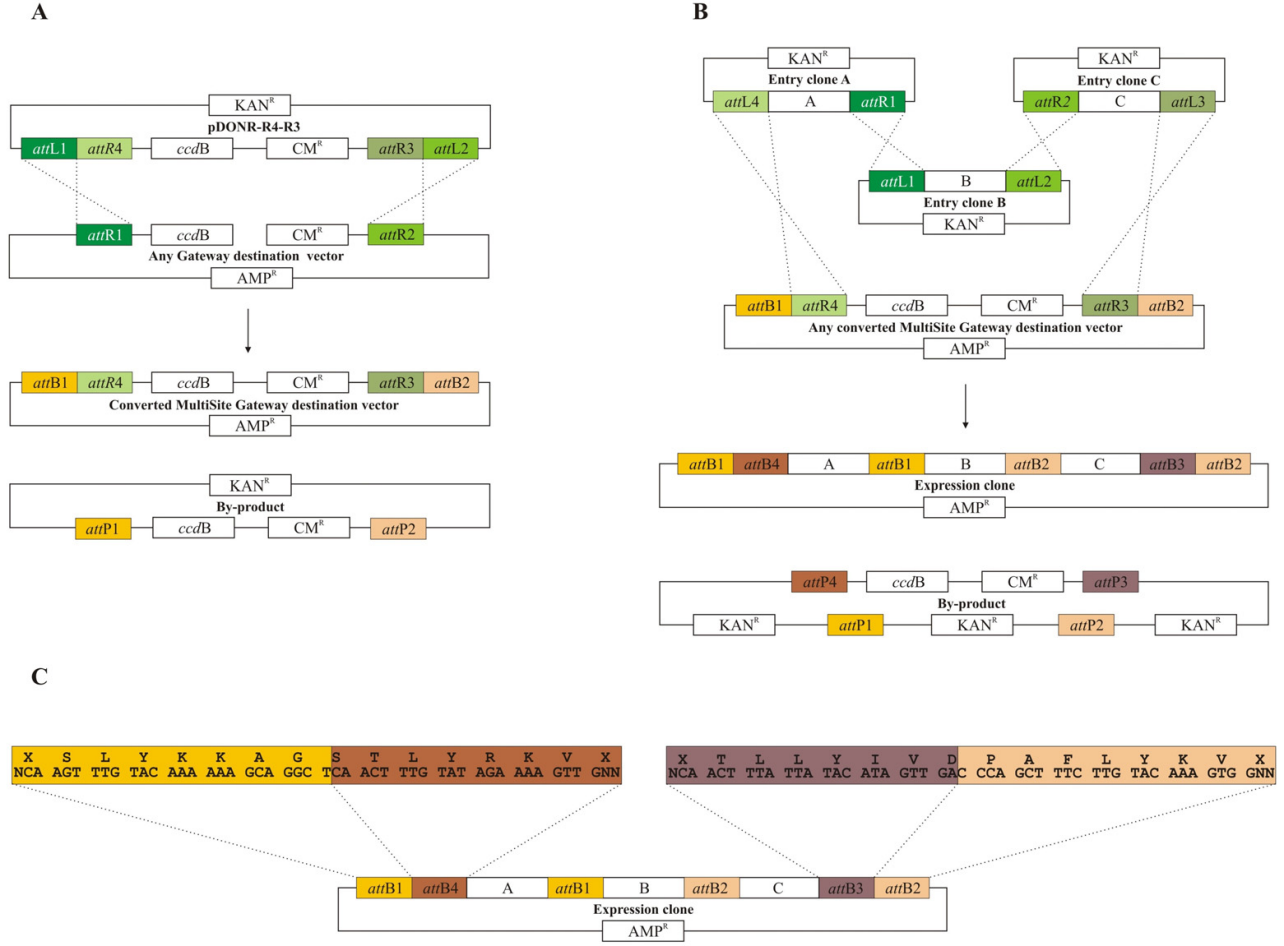
**Conversion of any Gateway destination vector to a MultiSite Gateway destination vector**. (**A**) LR recombination of the pDONR-R4-R3 vector with a linear Gateway destination vector. We tested this reaction using pDEST32 (Invitrogen) and pDEST22 (Invitrogen) destination vectors. (**B**) LR Plus recombination of three entry clones bearing the DNA fragments "A", "B" and "C" with a converted MultiSite Gateway destination vector. We tested this reaction by recombining three entry clones carrying a 3434 bp, 3116 bp and 561 bp DNA fragment with pDEST32-R4-R3 and pDEST22-R4-R3. (**C**) DNA and amino acid sequence of the external borders of the expression clone.

In order to use pDONR-R4-R3 to deliver the *att*R4-*att*R3 cassette into any Gateway destination vector in a single LR reaction, we took advantage of the fact that *att*R4 and *att*R3 sites cannot recombine with the flanking *att*L1 and *att*L2 sites (Fig 2B) [7]. To test it, we performed LR reactions with pDONR-R4-R3 and three different Gateway vectors: pDEST32 (Invitrogen), pDEST22 (Invitrogen) and pMDC163 [8]. These specific destination vectors were chosen to demonstrate the feasibility of the experiment. The linearization of pDEST32 and pDEST22 inside the Gateway cassette and their antibiotic resistance allowed us to specifically select for and recover the entry clones pDEST32-R4-R3 and pDEST22-R4-R3 bearing the *att*R4-*att*R3 cassette flanked by *att*B1-*att*B2 sites (Fig 4A). Some destination vectors, like pMDC163, carry the Kanamycin resistance gene in discordance with the Invitrogen recommendations. In this case, the linearization of the entry clone efficiently works as a negative selector against the non-recombined entry clone. The linearization of pDONR-R4-R3 in the vector backbone and pMDC163 inside the Gateway cassette allowed us to specifically recover pMDC163-R4-R3 (data not shown). The large number of Gateway vectors already available for various biological experiments can be easily converted to MultiSite Gateway vectors after an LR recombination with pDONR-R4-R3.

Last, we performed an LR Plus reaction with pDEST22-R4-R3 and pDEST32-R4-R3 to test the functionality of the *att*R4-*att*R3 cassette flanked by *att*B1-*att*B2 sites (Fig 4B). We used three entry clones bearing a 3434 bp, 3116 bp and 561 bp DNA fragment cloned into pDONR-P4-P1R (Invitrogen), pDONR221 and pDONR-P2R-P3 (Invitrogen) respectively [7]. Two LR Plus reactions were performed with the entry clones and either pDEST22-R4-R3 or pDEST32-R4-R3. Both reactions led to the creation of pDEST22 and pDEST32 expression clones containing the inserts in the expected order (Fig 4B). Correct functioning of the technology was further tested by cloning a different set of three entry clones into pDEST22-R4-R3 (data not shown). When the converted MultiSite Gateway destination vector bears a kanamycin resistance gene, like pMDC163-R4-R3, the entry clones need to be linearized in order to specifically recover the expression clone.

Figure 4C shows the external *att*B1-*att*B4 and *att*B3-*att*B2 sites of a converted MultiSite Gateway expression clone which maintain the Gateway frame and lack start and stop codons to allow promoter-gene or translational fusions. Thus, converted MultiSite Gateway vectors can take advantage of all reporter genes, tags or promoter sequences already present in the original Gateway destination vectors.

## Conclusion

We have developed a new method to convert virtually all Gateway vectors to MultiSite Gateway. A careful combination of compatible *att *sites allowed us to use the Gateway recombination technology to deliver a MultiSite Gateway cassette. This strategy opens a new way of thinking about recombination sites as cloning and clonable elements at the same time and could apply to other recombination cassettes. Our method makes the Invitrogen technology available to many biological systems for translational fusion of different proteins or tags, expression of genes or reporter genes under the control of specific promoters and 3'-UTRs, and combinatorial analysis of promoters, genes or other DNA fragments. Our improvement also expands the potential use of this recombination system by utilizing all the features of the large number of Gateway vectors already created.

## Methods

### Construction of pDONR-R4-R3

We PCR amplified the *att*R4-*att*R3 cassette from the pDEST-R4-R3 vector (Invitrogen) using the primers *att*B1-*att*R4-F (5'GGGGACAAGTTTGTACAAAAAAGCAGGCTCAACTTTGTATAGAAAAGTTGAAC3') and *att*B2-*att*R3-R (5'GGGGACCACTTTGTACAAGAAAGCTGGGTCAACTATGTATAATAAAGTTGAAC3'). We digested the pDONR221 vector (Invitrogen) with EcoRI and used it in a BP recombination reaction (Invitrogen) according to the manufacturer with the *att*R4-*att*R3 cassette PCR fragment. We used the reaction to transform DB3.1 *E. coli *cells (Invitrogen) and selected them for kanamycin resistance. We named the resultant vector pDONR-R4-R3.

### Construction of pDEST22-R4-R3, pDEST32-R4-R3 and pMDC163-R4-R3

We digested the pDEST22 (Invitrogen), pDEST32 (Invitrogen) and pMDC163 [8] vectors with EcoRI, XbaI and NcoI respectively. We conducted two independent LR reactions (Invitrogen) according to the manufacturer combining pDONR-R4-R3 with digested pDEST22 and pDEST32. An additional LR reaction was performed combining EcoNI linearized pDONR-R4-R3 with digested pMDC163. We used the three reactions to transform DB3.1 *E. coli *cells (Invitrogen) and selected them for ampicillin (pDEST22/pDONR-R4-R3 reaction), gentamicin (pDEST32/pDONR-R4-R3 reaction) or kanamycin (pMDC163/pDONR-R4-R3 reaction) resistance. We named the resultant vectors pDEST22-R4-R3, pDEST32-R4-R3 and pMDC163-R4-R3 respectively.

### Construction of pDONR-P4-P1R-pPNY, pDONR221-PNY and pDONR-P2R-P3-YC

We PCR amplified 3434 bp upstream the start codon of the Arabidopsis thaliana *PENNYWISE *(*PNY*) gene [9] using pny-*att*B4F (GGGGACAACTTTGTATAGAAAAGTTGTTGGCACGATTCTGAAACACG) and pny-*att*B1R (GGGGACTGCTTTTTTGTACAAACTTGGGGAAAGGATGATGTCGATGAG) primers, the *PNY *gene (3116 bp) using pny-*att*B1F (GGGACAAGTTTGTACAAAAAAGCAGGCTTGATGGCTGATGCATACGAGCC) and pny-*att*B2R (GGGGACCACTTTGTACAAGAAAGCTGGGTAACCTACAAAATCATGTAGAA) primers and a 561 bp fragment of the pUC-SPYCE vector [10] using *att*B2-SPYCE-F (GGGGACAGCTTTCTTGTACAAAGTGGGGATGTACCCATACGATGTTCCA) and *att*B3-SPY-R (GGGGACAACTTTGTATAATAAAGTTGGAATTCCCGATCTAGTAACATAGATG) primers. The *PNY *promoter region, the *PNY *gene and pUC-SPYCE PCR fragments were cloned into the pDONR-P4-P1R (Invitrogen), pDONR221 and pDONR-P2R-P3 (Invitrogen) vectors respectively via three independent BP reactions according to the manufacturer. We used the reactions to transform TOP10 *E. coli *cells (Invitrogen) and selected them for kanamycin resistance. We named the resultant vectors pDONR-P4-P1R-pPNY, pDONR221-PNY and pDONR-P2R-P3-YC respectively.

### Construction of pDEST22-pPNY-PNY-YC and pDEST32-pPNY-PNY-YC

We performed an LR Plus reaction (Invitrogen) according to the manufacturer combining pDONR-P4-P1R-pPNY, pDONR221-PNY, pDONR-P2R-P3-YC, and pDEST22-R4-R3. We used the reaction to transform TOP10 *E. coli *cells and selected for ampicillin resistance. We named the resultant vectors pDEST22-pPNY-PNY-YC. We performed another LR Plus reaction according to the manufacturer combining pDONR-P4-P1R-pPNY, pDONR221-PNY, pDONR-P2R-P3-YC, and pDEST32-R4-R3. We used the reaction to transform TOP10 *E. coli *cells and selected for gentamicin resistance. We named the resultant vectors pDEST32-pPNY-PNY-YC.

## List of abbreviations

AMP^r^, ampicillin resistance.

CM^R^, chloramphenicol resistance.

KAN^R^, kanamycin resistance.

## Competing interests

The author(s) declare that they have no competing interests.

## Authors' contributions

EM conceived and designed the study, performed the experiments and drafted the manuscript. LB created the entry clones, performed one LR plus reaction and sequenced the final expression clones. SH helped draft the manuscript. All authors read and approved the final manuscript.
